# Pre-Eclampsia Increases the Risk of Postpartum Haemorrhage: A Nationwide Cohort Study in The Netherlands

**DOI:** 10.1371/journal.pone.0081959

**Published:** 2013-12-18

**Authors:** Joost F. von Schmidt auf Altenstadt, Chantal W. P. M. Hukkelhoven, Jos van Roosmalen, Kitty W. M. Bloemenkamp

**Affiliations:** 1 Department of Obstetrics, Leiden University Medical Centre, Leiden, The Netherlands; 2 The Netherlands Perinatal Registry, Utrecht, The Netherlands; 3 Department of Medical Humanities, EMGO Institute for Health and Care Research, VU University Medical Centre, Amsterdam, The Netherlands; Yale School of Public Health, United States of America

## Abstract

**Background:**

Postpartum haemorrhage is a leading cause of maternal morbidity and mortality worldwide. Identifying risk indicators for postpartum haemorrhage is crucial to predict this life threatening condition. Another major contributor to maternal morbidity and mortality is pre-eclampsia. Previous studies show conflicting results in the association between pre-eclampsia and postpartum haemorrhage. The primary objective of this study was to investigate the association between pre-eclampsia and postpartum haemorrhage. Our secondary objective was to identify other risk indicators for postpartum haemorrhage in the Netherlands.

**Methods:**

A nationwide cohort was used, containing prospectively collected data of women giving birth after 19 completed weeks of gestation from January 2000 until January 2008 (n =  1 457 576). Data were extracted from the Netherlands Perinatal Registry, covering 96% of all deliveries in the Netherlands. The main outcome measure, postpartum haemorrhage, was defined as blood loss of ≥1000 ml in the 24 hours following delivery. The association between pre-eclampsia and postpartum haemorrhage was investigated with uni- and multivariable logistic regression analyses.

**Results:**

Overall prevalence of postpartum haemorrhage was 4.3% and of pre-eclampsia 2.2%. From the 31 560 women with pre-eclampsia 2 347 (7.4%) developed postpartum haemorrhage, compared to 60 517 (4.2%) from the 1 426 016 women without pre-eclampsia (odds ratio 1.81; 95% CI 1.74 to 1.89). Risk of postpartum haemorrhage in women with pre-eclampsia remained increased after adjusting for confounders (adjusted odds ratio 1.53; 95% CI 1.46 to 1.60).

**Conclusion:**

Women with pre-eclampsia have a 1.53 fold increased risk for postpartum haemorrhage. Clinicians should be aware of this and use this knowledge in the management of pre-eclampsia and the third stage of labour in order to reach the fifth Millenium Developmental Goal of reducing maternal mortality ratios with 75% by 2015.

## Introduction

Postpartum haemorrhage (PPH) accounts for 25–35% of all maternal deaths worldwide. [1], [2] Unfortunately, varying definitions exist. The World Health Organization defines PPH as blood loss ≥500 ml in the first 24 hours after delivery, although in high resources settings a definition of ≥1000 ml blood loss seems more suitable. [3]–[6] Main causes of PPH are uterine atony, retained placenta, maternal soft tissue trauma and coagulopathy. [7]–[9]

Even though in high income countries the maternal mortality ratio is much lower than in low income countries, still 13% results from PPH. [2] Because of low maternal mortality ratios severe acute maternal morbidity has gained interest as a new quality indicator of obstetric care. In a nationwide cohort study in the Netherlands the incidence of major obstetric haemorrhage was 4.5 per 1000 deliveries and the case fatality rate was 1:201 (0.5%). [10] Prevention and management of PPH is crucial in order to reach the fifth Millennium Developmental Goal to reduce maternal mortality ratios with 75% by 2015. [1] This emphasises the importance of identifying specific risk indicators, such as previous PPH, multiple pregnancies, macrosomia, induction of labour, prolonged labour, operative vaginal deliveries and caesarean section. [8], [11]–[18] Another major contributor to maternal death and morbidity is pre-eclampsia (PE), complicating 2–8% of pregnancies worldwide and 2–5% in high resource countries. [2], [19], [20] While multiple studies investigated PE as one of many risk indicators for PPH [8], [11], [12], [15], [16], [21], [22] only one study focused primarily on the association between PE and PPH. [21] This is conceivable considering its multifactorial pathogenesis, where angiogenic factors, endothelial dysfunction and impaired uteroplacental blood flow result in hypertension and coagulation abnormalities. [23]

Clarification of the association between the two most important causes of maternal mortality and morbidity will help improve their prevention and management. Therefore, our objective was to investigate the association between PE and PPH. Our secondary objective was to determine the prevalence and risk indicators for PPH among pregnant women in the Netherlands.

## Materials and Methods

### Database

We used data from the Netherlands Perinatal Registry, a linked nationwide registry that includes maternal, obstetric, postpartum and neonatal information of each delivery from January 2000 until January 2008. [24]–[26] Approximately 96% of all births in the Netherlands are entered into this registry.

### Definitions

Main outcome measure, PPH, was defined as blood loss of ≥1000 ml in the 24 hours following delivery. PE was defined as a minimum diastolic blood pressure of 90 mmHg with the presence of proteinuria after 20 weeks of gestation, according to the recommendation of the International Society for the Study of Hypertension in Pregnancy. [27] The registration form contains a field for maximum diastolic blood pressure and an option for proteinuria "yes" or "no" with a field for the amount of proteinuria. The definition of PE was based on the presence of proteinuria rather than ≥0.3 g/day, since 24.0% of women with proteinuria had a missing field for "amount of proteinuria". Women in primary care are referred to secondary care if proteinuria is present or diastolic blood pressure is over 95 mmHg or if the combination of more than 90 mmHg with complaints exists. In primary care all women with a diastolic blood pressure of 90 mmHg are checked more frequently and controlled for proteinuria according to existing guidelines. [28] In addition we defined severe PE as eclampsia or PE with blood pressure ≥110 mmHg or proteinuria ≥5 grams or gestational age of less than 32 weeks.

### Characteristics

We evaluated maternal, pregnancy, labour and postpartum characteristics. Maternal characteristics included age at the time of delivery, categorised into six groups: <20, 20–24, 25–29, 30–34, 35–39 or ≥40 years. Parity was grouped as either nulliparous, parous (para 1–4), or grand multiparous (para ≥5). Ethnicity included the categories European descent and non-European descent, in which the options Dutch and other European represented the European descent group. Socioeconomic status was categorised into high, middle or low by using mean household income levels of a neighborhood, which was determined with the first four digits of the postal code, using data from the Netherlands Institute for Social Research. [29]

Pregnancy characteristics were multiple pregnancy (defined as "yes" or "no"), and gestational age, categorised as early preterm (≤31 weeks), late preterm (32–36 weeks), term (37–41 weeks) or post term (≥42 weeks) birth.

Labour characteristics included fetal presentation, defined as vertex, breech or other (transverse, face and other presentations). Onset of labour was defined as whether or not induction or elective caesarean section was performed. Furthermore, duration of ruptured membranes was grouped as < or ≥12 hours. We defined augmented labour as "yes" or "no" and prolonged expulsive phase of labour as < or ≥60 minutes. Mode of delivery was categorised as spontaneous delivery, assisted vaginal delivery (vacuum, forcipal or breech extraction), elective (planned) or emergency (unplanned) caesarean section. In the Netherlands, caesarean sections are recorded differently (planned/unplanned) compared to other countries (elective/emergency). Our system is based on an intention to treat mechanism, implying that a woman is identified as planned (elective) if she was intended to deliver by caesarean, even if she presents in labour. [30] Genital tract injury was defined as perineal tear, episiotomy or both. First degree perineal tears were not classified as genital tract injury. We classified the use of anaesthetics hierarchically, considering the possibility of receiving different types during delivery. Options were: no or primary care medication (non-opioid analgesics and sedatives), opioids, epidural during labour, epidural or spinal anaesthesia at caesarean section or general anaesthesia. If a woman had been given multiple options she was assigned to the highest category.

Postpartum characteristics consisted of manual placenta removal ("yes" or "no") and birth weight, categorised into ≤999, 1000–1999, 2000–2999, 3000–3999 or ≥4000 gram.

### Statistical analysis

Contingency tables were created to assess frequencies and percentages of the characteristics and the main outcome measure among women with and without PE. Differences were tested with χ^2^-statistics. The characteristics of the excluded women with missing values on PE or PPH were investigated. Univariable logistic regression analysis was performed on all characteristics to identify risk indicators for PPH. The reference category of each variable represented the largest number of women in the population.

The association between (severe) PE and PPH was studied with uni- and multivariable logistic regression, adjusting for maternal age, parity, ethnicity, socioeconomic status, multiple pregnancy, and gestational age. All these indicators have a pathofysiologic explanation for confounding the association of PE with PPH. [31], [32] We also assessed presence of effect modification between multiple pregnancy and PE using the likelihood-ratio test.

Even though mode of delivery and induction of labour are important variables in the association of PE and PPH, they are intermediate factors in the causal pathway of the association and were therefore not considered as confounders. [31], [32] For that reason we performed subgroup analysis on women with non-induced spontaneous delivery and investigated the other subgroups for the association between PE and PPH [33].

Seeing an increasing trend of PPH over time the International Postpartum Hemorrhage Collaborative Group recommends countries to publish its yearly prevalence. [6], [17], [18], [34] Also the prevalence of PE has been shown to increase, therefore we analysed yearly prevalences of both PE and PPH. [20] We performed Cochran-Armitage and Jonckheere-Terpstra tests to evaluate possible trends.

Statistical analysis was performed using SAS (version 9.3; SAS Institute, Cary, NC).

### Ethics statement

The presented data are anonymised and cannot be related to individual women. The privacy committee of the Netherlands Perinatal Registry approved this study. Further consent and ethical approval is not needed in the Netherlands for this type of study.

## Results

During our study period 1 621 053 women delivered above 19 completed weeks of gestation. After excluding missing values our study population comprised 1 457 576 women (excluded were 108 478 missing PE, 47 885missing PPH and 7 114 missing PE and PPH; respectively 6.7%, 2.9% and 0.4% of the total population). Tables 1, 2, and 3 show the prevalence of maternal, pregnancy, labour and postpartum characteristics among all women and women with and without PE or PPH, together with corresponding odds ratios (ORs) for PPH.

**Table 1:** Maternal and pregnancy characteristics of the study population (n = 1.457.576) and the association between these characteristcs and postpartum haemorrhage.

		Pre-eclampsia				Postpartum haemorrhage						Unadjusted odds	
		Yes*		No*		Yes*		No*		Total*		ratio for postpartum haemorrhage	
Characteristic		n	(%)	n	(%)	n	(%)	n	(%)	n	(%)	OR	(95% CI)
**Pregnant women**		31560	(2.2)	1426016	(97.8)	62864	(4.3)	1394712	(95.7)	1457576	(100.0)		
**Maternal characteristics**													
Maternal age, years†													
	≤19	535	(1.7)	23340	(1.6)	770	(1.2)	23105	(1.7)	23875	(1.6)	0.70	(0.66 to 0.75)
	20–24	3519	(11.2)	143912	(10.1)	5149	(8.2)	142282	(10.2)	147431	(10.1)	0.76	(0.74 to 0.78)
	25–29	9841	(31.2)	411110	(28.8)	17163	(27.3)	403788	(29.0)	420951	(28.9)	0.90	(0.88 to 0.92)
	30–34	11332	(35.9)	563918	(39.6)	25903	(41.2)	549347	(39.4)	575250	(39.5)	1.00	reference
	35–39	5342	(16.9)	248610	(17.4)	12091	(19.2)	241861	(17.3)	253952	(17.4)	1.07	(1.04 to 1.09)
	≥40	989	(3.1)	34907	(2.4)	1780	(2.8)	34116	(2.4)	35896	(2.5)	1.13	(1.08 to 1.19)
	Missing	2		219		8		213		221			
Parity†													
	0	22006	(69.7)	643195	(45.1)	32592	(51.9)	632609	(45.4)	665201	(45.6)	1.27	(1.25 to 1.29)
	1–4	9338	(29.6)	770926	(54.1)	29783	(47.4)	750481	(53.8)	780264	(53.5)	1.00	reference
	≥5	214	(0.7)	11762	(0.8)	481	(0.8)	11495	(0.8)	11976	(0.8)	1.07	(0.98 to 1.16)
	Missing	2		133		8		127		135			
Ethnicity (European descent)‡													
	No	5002	(15.9)	216407	(15.3)	8914	(14.2)	212495	(15.3)	221409	(15.3)	0.90	(0.88 to 0.92)
	Yes	26475	(84.1)	1200916	(84.7)	53712	(85.8)	1173679	(84.7)	1227391	(84.7)	1.00	reference
	Missing	83		8693		238		8538		8776			
Socioeconomic status†													
	High	6812	(21.9)	333703	(23.7)	15720	(25.4)	324795	(23.6)	340515	(23.7)	1.09	(1.07 to 1.11)
	Medium	14069	(45.2)	647662	(46.0)	28165	(45.5)	633566	(46.0)	661731	(46.0)	1.00	reference
	Low	10258	(32.9)	426359	(30.3)	18071	(29.2)	418546	(30.4)	436617	(30.3)	0.97	(0.95 to 0.98)
	Missing	421		18292		908		17805		18713			
**Pregnancy characteristics**													
Multiple pregnancy†													
	No	29446	(93.3)	1400362	(98.2)	59492	(94.6)	1370316	(98.3)	1429808	(98.1)	1.00	reference
	Yes	2114	(6.7)	25654	(1.8)	3372	(5.4)	24396	(1.7)	27768	(1.9)	3.07	(2.96 to 3.17)
Gestational age, weeks†													
	≤31^+6^	2277	(7.3)	14846	(1.0)	1360	(2.2)	15763	(1.1)	17123	(1.2)	2.01	(1.91 to 2.11)
	32^+0^ – 36^+6^	8086	(25.8)	70628	(5.0)	4435	(7.1)	74279	(5.4)	78714	(5.4)	1.37	(1.33 to 1.41)
	37^+0^ – 41^+6^	20680	(65.9)	1262219	(89.0)	52339	(83.7)	1230560	(88.7)	1282899	(88.5)	1.00	reference
	≥42^+0^	342	(1.1)	70116	(4.9)	4409	(7.0)	66049	(4.8)	70458	(4.9)	1.54	(1.49 to 1.59)
	Missing	175		8207		321		8061		8382			

Missing values of postpartum haemorrhage and pre-eclampsia are exluded.

^2^test between pre-eclampsia and no pre-eclampsia, P ≤ 0.0001. X

^2^test between pre-eclampsia and no pre-eclampsia,, P =  0.01. X

**Table 2:** Labour characteristics of the study population (n = 1.457.576) and the association between these characteristcs and postpartum haemorrhage.

		Pre-eclampsia				Postpartum haemorrhage						Unadjusted odds ratio for	
		Yes*		No*		Yes*		No*		Total*		postpartum haemorrhage	
Characteristic		n	(%)	N	(%)	n	(%)	n	(%)	n	(%)	OR	(95% CI)
**Pregnant women**		31560	(2.2)	1426016	(97.8)	62864	(4.3)	1394712	(95.7)	1457576	(100.0)		
Presentation†													
	Vertex	28405	(90.1)	1346629	(94.5)	59701	(95.0)	1315333	(94.3)	1375034	(94.4)	1.00	reference
	Breech	2742	(8.7)	69527	(4.9)	2496	(4.0)	69773	(5.0)	72269	(5.0)	0.80	(0.77 to 0.83)
	Other	382	(1.2)	9327	(0.7)	618	(1.0)	9091	(0.7)	9709	(0.7)	1.54	(1.42 to 1.66)
	Missing	31		533		49		515		564			
Onset of labour†													
	Non-induced labour	6700	(21.3)	1146966	(80.5)	46120	(73.6)	1107546	(79.5)	1153666	(79.2)	1.00	reference
	Induced labour	17438	(55.4)	193983	(13.6)	12945	(20.6)	198476	(14.2)	211421	(14.5)	1.52	(1.49 to 1.55)
	Elective caesarean	7334	(23.3)	83639	(5.9)	3624	(5.8)	87349	(6.3)	90973	(6.2)	1.02	(0.99 to 1.06)
	Missing	88		1428		175		1341		1516			
Ruptured membranes, hours†													
	<12	25617	(86.1)	1134348	(82.9)	47867	(78.5)	1112098	(83.2)	1159965	(83.0)	1.00	reference
	≥12	4132	(13.9)	233257	(17.1)	13117	(21.5)	224272	(16.8)	237389	(17.0)	1.35	(1.32 to 1.37)
	Missing	1811		58411		1880		58342		60222			
Augmentation of labour†													
	No	22931	(72.7)	1141004	(80.0)	45180	(71.9)	1118755	(80.2)	1163935	(79.9)	1.00	reference
	Yes	8629	(27.3)	285012	(20.0)	17684	(28.1)	275957	(19.8)	293641	(20.1)	1.52	(1.50 to 1.55)
Expulsive phase, minutes†													
	<60	27553	(88.0)	1186426	(84.0)	48349	(77.8)	1165630	(84.4)	1213979	(84.1)	1.00	reference
	≥60	3767	(12.0)	225148	(16.0)	13780	(22.2)	215135	(15.6)	228915	(15.9)	1.52	(1.49 to 1.55)
	Missing	240		14442		735		13947		14682			
Mode of delivery†													
	Spontaneous delivery	14784	(46.9)	1088924	(76.4)	46376	(74.0)	1057332	(75.9)	1103708	(75.8)	1.00	reference
	Assisted delivery	3959	(12.6)	144854	(10.2)	9383	(15.0)	139430	(10.0)	148813	(10.2)	1.49	(1.46 to 1.52)
	Elective caesarean	7334	(23.3)	83639	(5.9)	3624	(5.8)	87349	(6.3)	90973	(6.2)	0.98	(0.95 to 1.08)
	Emergency caesarean	5453	(17.3)	107304	(7.5)	3275	(5.2)	109482	(7.9)	112757	(7.7)	0.72	(0.70 to 0.74)
	Missing	30		1295		206		1119		1325			
Genital tract injury†													
	Episiotomy (no)/perineal tear (no)	23376	(74.1)	1024303	(71.9)	37681	(60.0)	1009998	(72.4)	1047679	(71.9)	1.00	reference
	Episiotomy (no)/perineal tear (yes)	364	(1.2)	23953	(1.7)	1679	(2.7)	22638	(1.6)	24317	(1.7)	1.92	(1.83 to 2.01)
	Episiotomy (yes)/perineal tear (no)	7714	(24.5)	371807	(26.1)	22933	(36.5)	356588	(25.6)	379521	(26.0)	1.69	(1.66 to 1.71)
	Episiotomy (yes)/perineal tear (yes)	84	(0.3)	5473	(0.4)	522	(0.8)	5035	(0.4)	5557	(0.4)	2.68	(2.46 to 2.92)
	Missing	22		480		49		453		502			
Anaesthesia†													
	No/primary care medication	13797	(43.7)	1075885	(75.4)	45774	(72.8)	1043908	(74.8)	1089682	(74.8)	1.00	reference
	Opioid analgesics	3449	(10.9)	104489	(7.3)	5939	(9.4)	101999	(7.3)	107938	(7.4)	1.28	(1.24 to 1.31)
	Epidural during labour	2892	(9.2)	77721	(5.5)	5219	(8.3)	75394	(5.4)	80613	(5.5)	1.27	(1.24 to 1.31)
	Spinal or epidural at caesarean	8795	(27.9)	151714	(10.6)	4970	(7.9)	155539	(11.2)	160509	(11.0)	0.75	(0.73 to 0.77)
	General anaesthesia	2627	(8.3)	16207	(1.1)	962	(1.5)	17872	(1.3)	18834	(1.3)	1.32	(1.24 to 1.40)

Missing values of postpartum haemorrhage and pre-eclampsia are excluded

^2^test between pre-eclampsia and no pre-eclampsia, P ≤ 0.0001 X

**Table 3:** Postpartum characteristics of the study population (n = 1.457.576) and the association between these characteristcs and postpartum haemorrhage.

		Pre-eclampsia				Postpartum haemorrhage						Unadjusted odds ratio for	
		Yes*		No*		Yes*		No*		Total*		postpartum haemorrhage	
Characteristic		n	(%)	n	(%)	n	(%)	n	(%)	n	(%)	OR	(95% CI)
**Pregnant women**		31560	(2.2)	1426016	(97.8)	62864	(4.3)	1394712	(95.7)	1457576	(100.0)		
**Postpartum characteristics**													
Manual placenta removal†													
	No	30348	(96.3)	1389321	(98.1)	47128	(75.2)	1372541	(99.1)	1419669	(98.1)	1.00	reference
	Yes	1181	(3.7)	26759	(1.9)	15525	(24.8)	12415	(0.9)	27940	(1.9)	33.39	(32.62 to 34.19)
	Missing	31		9936		211		9756		9967			
Birthweight, grams†													
	≤999	1183	(3.7)	9013	(0.6)	832	(1.3)	9364	(0.7)	10196	(0.7)	2.08	(1.96 to 2.23)
	1000 – 1999	4639	(14.7)	17882	(1.3)	1259	(2.0)	21262	(1.5)	22521	(1.5)	1.48	(1.41 to 1.56)
	2000 – 2999	12035	(38.1)	230514	(16.2)	9663	(15.4)	232886	(16.7)	242549	(16.6)	1.01	(0.99 to 1.03)
	3000 – 3999	11604	(36.8)	942823	(66.1)	37305	(59.4)	917122	(65.8)	954427	(65.5)	1.00	reference
	≥4000	2097	(6.6)	225442	(15.8)	13776	(21.9)	213763	(15.3)	227539	(15.6)	1.59	(1.56 to 1.62)
	Missing	2		342		29		315		344			

Missing values of postpartum haemorrhage and pre-eclampsia are excluded.

^2^test between pre-eclampsia and no pre-eclampsia, P ≤ 0.0001. X

Overall prevalence of PE was 2.2%. Women with PE were more often nulliparous, had more often multiple pregnancies and had lower gestational age at birth (table 1). Furthermore, women with PE more often had elective caesarean section, induction and/or augmentation of labour, a shorter expulsive phase and less often delivered spontaneously (table 2). The groups also differed significantly in genital tract injury, anaesthetic use and manual placenta removal (tables 2 and 3).

In our study population 4.3% of the women suffered from PPH (table 1). From the 31 560 women with PE 2 347 (7.4%) developed PPH, compared to 60 517 (4.2%) from the 1 426 016 women without PE (OR 1.81; 95% CI 1.74 to 1.89, p<0.001).

The group women with missing values for PE contained more PPH (6.6% compared with 4.3% in the total population), while missing values for PPH showed slightly more PE (2.5% compared with 2.2% in the total population). Investigating the missing values showed no clear tendency for either PPH or PE. Therefore missing data is not expected to be adherent to either the exposure or the outcome measure; the missing data is assumed to be randomly distributed [35]

Table 4 shows the adjusted association between PE and PPH in all women and in a subgroup of women with non-induced spontaneous delivery. Univariably, PE increased the risk of PPH 1.81 fold (95% CI 1.74 to 1.89). After adjustment for confounders we observed a slightly lower but still statistically significant increased risk for PPH (OR 1.53, 95% CI 1.46 to 1.60). In this model non-European descent ethnicity and low socioeconomic status lost their statistical significance. In the subgroup analysis of women with non-induced spontaneous delivery PE increased the risk for PPH (adjusted OR 1.91, 95% CI 1.71 to 2.13; other subgroup analyses are shown in footnotes table 4). Distinguishing the severity of PE showed women with mild PE have a higher increased risk for PPH (adjusted OR 1.67; 95% CI 1.58 to 1.77) than women with severe PE (adjusted OR 1.32; 95% CI 1.23 to 1.42), while the subgroup of non-induced spontaneous deliveries showed reversed results (mild PE, adjusted OR 1.76, 95% CI 1.55 to 2.01; severe PE, adjusted OR 2.36; 95% CI 1.92 to 2.88).

**Table 4:** Multivariate analysis on the association between pre-eclampsia and postpartum haemorrhage and subgroup of women with non-induced spontaneous delivery.

		Total cohort		Subgroup non-induced spontaneous delivery†	
		Adjusted odds ratio for postpartum haemorrhage*		Adjusted odds ratio for postpartum haemorrhage*	
Risk indicator		OR	(95% CI)	OR	(95% CI)
Pre-eclampsia					
	No	1.00	reference	1.00	reference
	Yes	1.53	(1.46 to 1.60)	1.91	(1.71 to 2.13)
Maternal age, years					
	≤19	0.62	(0.58 to 0.67)	0.63	(0.57 to 0.68)
	20–24	0.71	(0.69 to 0.73)	0.71	(0.68 to 0.74)
	25–29	0.86	(0.84 to 0.88)	0.85	(0.83 to 0.88)
	30–34	1.00	reference	1.00	reference
	35–39	1.09	(1.07 to 1.11)	1.08	(1.05 to 1.12)
	≥40	1.15	(1.09 to 1.21)	1.13	(1.05 to 1.21)
Parity					
	0	1.36	(1.34 to 1.39)	1.48	(1.45 to 1.51)
	1–4	1.00	reference	1.00	reference
	≥5	0.95	(0.87 to 1.05)	0.85	(0.75 to 0.97)
Ethnicity					
	European descent	1.00	reference	1.00	reference
	Non-European descent	1.02	(0.99 to 1.04)	1.04	(1.01 to 1.07)
Socioeconomic status					
	High	1.07	(1.05 to 1.09)	1.08	(1.05 to 1.11)
	Medium	1.00	reference	1.00	reference
	Low	1.00	(0.98 to 1.02)	1.01	(0.99 to 1.04)
Multiple pregnancy					
	No	1.00	reference	1.00	reference
	Yes	2.88	(2.77 to 3.00)	2.36	(2.18 to 2.54)
Gestational age, weeks					
	≤31^+6^	1.53	(1.44 to 1.62)	2.15	(1.98 to 2.34)
	32^+0^ – 36^+6^	1.05	(1.02 to 1.09)	1.16	(1.11 to 1.22)
	37^+0^ – 41^+6^	1.00	reference	1.00	reference
	≥42^+0^	1.56	(1.51 to 1.61)	1.68	(1.58 to 1.79)

Adjusted for pre-eclampsia, maternal age, parity, ethnicity, socioeconomic status, multiple pregnancy and gestational age.

% CI; 1.68 to 2.44). Subgroup non-induced emergency caesarean section OR 1.55 (95% CI; 1.16 to 2.06). Subgroup induced spontaneous delivery OR 1.46 (95% CI; 1.36 to 1.58). Subgroup induced assisted delivery OR 1.61 (95% CI; 1.41 to 1.84). Subgroup induced emergency caesarean section OR 1.40 (95% CI; 1.17 to 1.68). Subgroup elective caesarean section OR 0.98 (95% CI; 0.86 to 1.12). Adjusted risk of pre-eclampsia for postpartum haemorrhage in other subgroups: Subgroup non-induced assisted delivery OR 2.02 (95

We found no evidence of effect modification between PE and multiple pregnancy.

**Figure 1:**
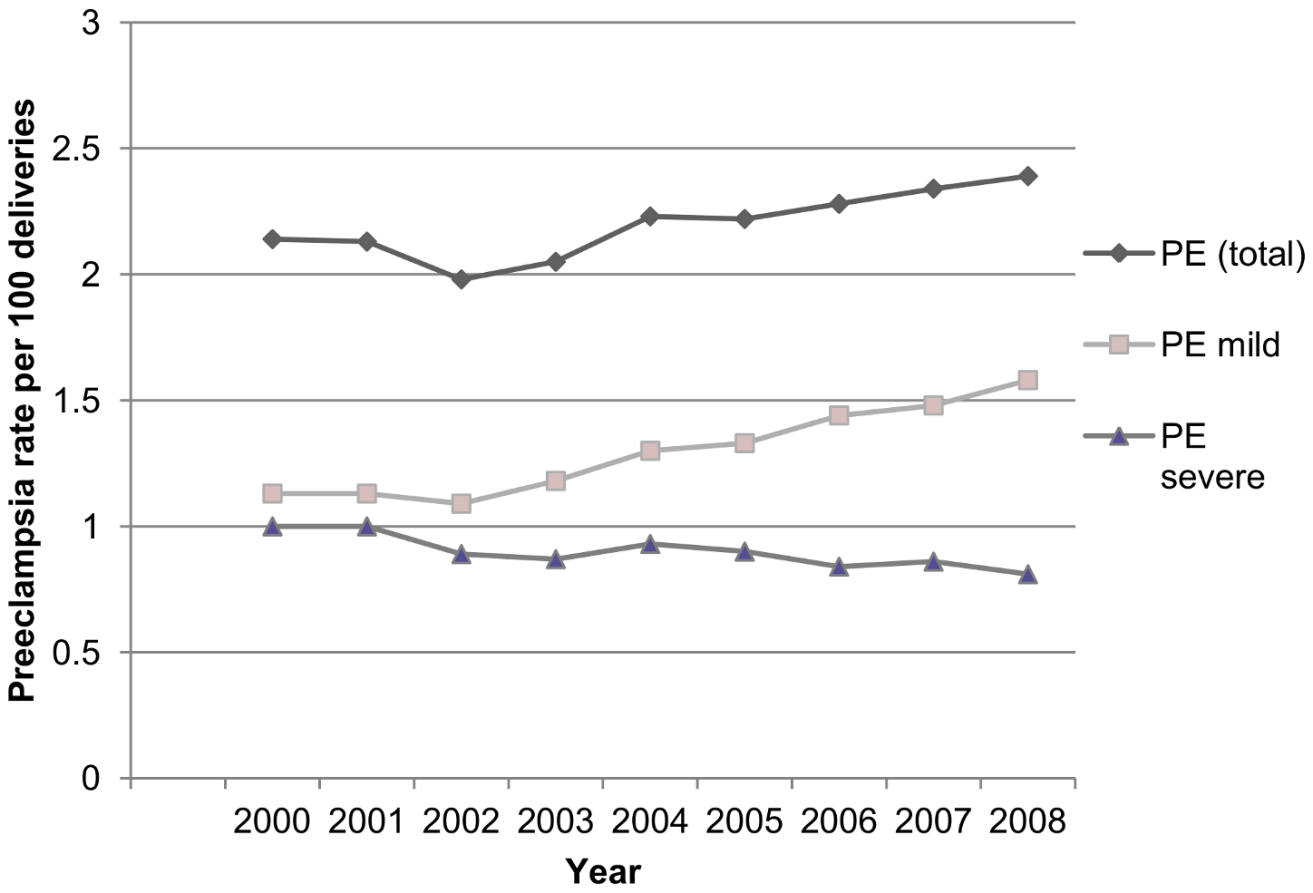
Annual rates of pre-eclampsia (PE), 2000–2008.

**Figure 2:**
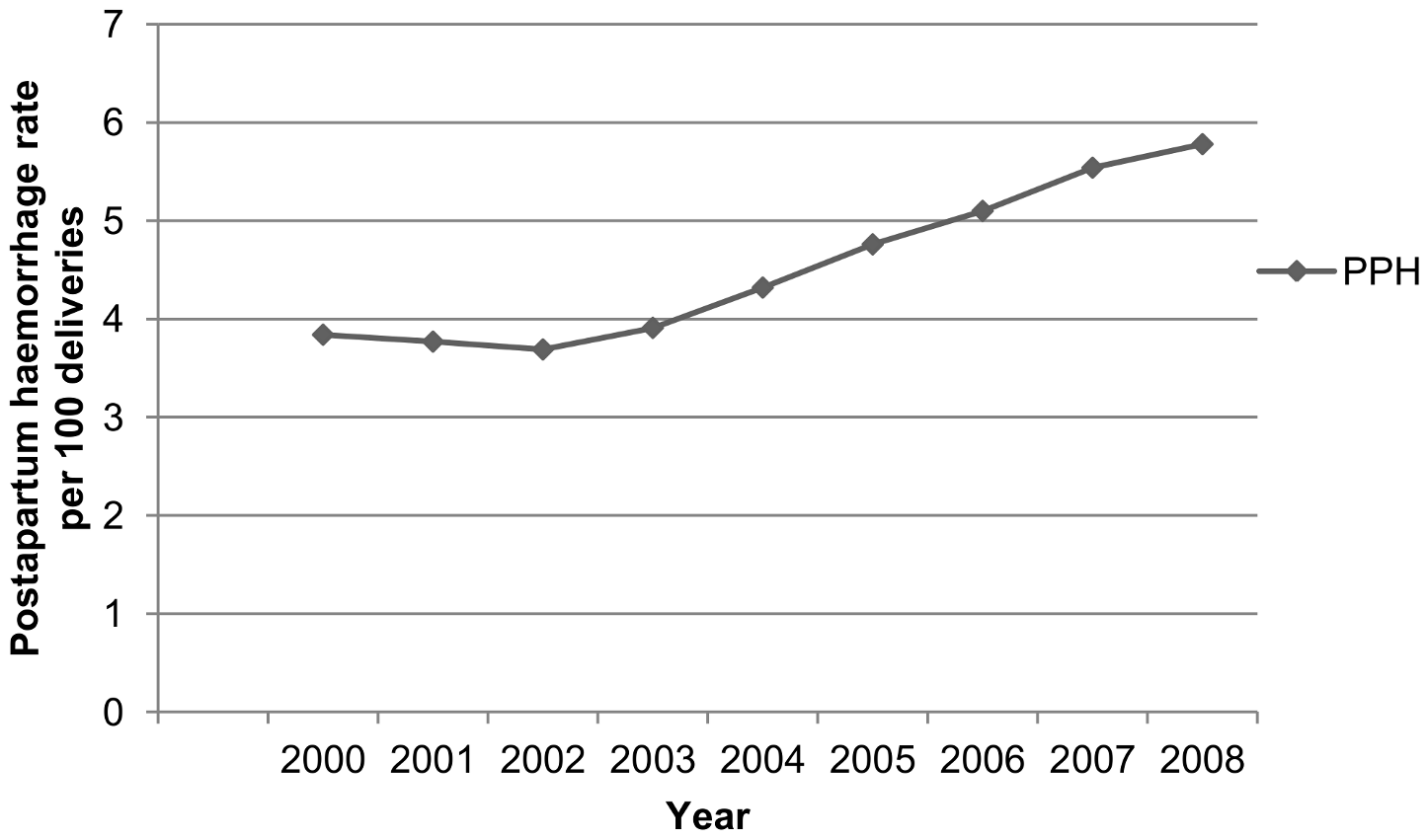
Annual rates of postpartum haemorrhage (PPH), 2000–2008.

Figure 1 and 2 show the yearly percentages of PE (total, mild and severe) and PPH. There is a statistically significant increase of PE (P<0.0001) and PPH (P<0.0001) over time. We observed a rising trend of mild PE, while the proportion of severe PE gradually declined (P<0.0001).

## Discussion

### Principal findings

Our study shows an adjusted 1.53 fold increased risk (95% CI 1.46 to 1.60) for developing PPH in women with PE.

Women with PE were more often older, nulliparous, had multiple pregnancies, a lower gestational age and a lower socioeconomic status. These indicators are also associated with increased risk for PPH, which could explain a slight decrease in risk for PPH after adjustment for confounders. [8], [11]–[15], [22], [36]–[39] Remarkably non-induced women with PE who delivered vaginally revealed an even stronger adjusted association with PPH (adjusted OR 2.29, 95% CI 2.06 to 2.55). Except for women undergoing elective caesarean section all other subgroups showed women with PE had an increased risk for PPH, suggesting the association is independent for onset of labour or mode of delivery. The observed shift in risk when assessing the severity of PE could indicate a positive side-effect in the management of PE; induction possibly reduces the risk for PPH in women with severe PE.

Our nationwide study population had a prevalence of 2.2% for PE and 4.3% for PPH, similar rates are seen internationally (United States [20], Canada [17], [40]–[42], Scandinavia [43]–[45], United Kingdom [46], Australia [47], [48]). A worrying finding is the rising trend of both PE and PPH. The increase in PE could be explained by a rising prevalence of risk indicators for PE, e.g. high body mass index, hypertension, diabetes mellitus, high maternal age. [17], [18], [20], [34], [49] The decreasing trend of severe PE may be attributed to a more aggressive management of PE over the years. The increase in PPH has been studied in many other high resource countries. [6], [17], [18], [34] They state this finding could be explained by an increase in risk indicators (e.g. maternal age, multiple pregnancies, induction of labour, caesarean section). However adjustment for these indicators did not explain the observed trend. [17], [18], [34] Another possibility could be an increasing awareness for PPH with subsequently better recognition and registration.

### Strengths and weaknesses of the study

A strength of this study is the large population based database with a nationwide and near complete inclusion of all deliveries with detailed information on maternal, pregnancy, labour and postpartum characteristics. Despite the retrospective design data were prospectively collected. To our knowledge this is the largest cohort study on the relation between PE and PPH.

Another positive aspect of this study is the fact that our definitions for PE and PPH are not based on International Classification Disease (ICD) codes. Inaccuracies may occur when translating definitions to ICD-codes, since they are not thoroughly defined. ICD-codes neglect to take amount of blood loss, blood pressure or amount of proteïnuria into account, while diseases may be defined differently between countries, regions or even hospitals. [6] Therefore registration is not unified and coding might be subject to clinical interpretation of the clinician. This also presents our first limitation of the study. Even though our definition is derived from obligated objective items, we had to base the definition of PPH on estimation of blood loss by the caregiver, either weighted or visual. Visual estimation has been shown to underestimate blood loss, suggesting a higher prevalence of PPH than the observed 4.3%. [5], [50], [51] Furthermore, our overall definition of PE was based on the presence of proteinuria rather than a measured amount. Since dipstick urinalysis of protein is not 100% accurate, [52] we performed a sensitivity analysis in women with a known amount of proteinuria, now defining PE with the amount of proteinuria instead of the presence (≥0.3 g/day). This showed robustness of our findings (adjusted OR 1.49; 95% CI 1.42 to 1.57). Caution is justified in interpreting our results based on severity of PE. As there is a high percentage of missing values for proteinuria some severe PE cases could have been misclassified as mild. Yet other studies show similar results in the observed trends. [20]

Another limitation is the 6.7% women whose information on PE was missing. Characteristics of these women did not clearly correspond to either the PE or the non-PE group. A biased estimation of the association between PE and PPH is not expected, as complete case analysis with adjustment for covariates covering randomly distributed missing data (MAR), has been shown to estimate unbiased results. [35]

The perinatal registry does not contain valid or complete information on body mass index, previous PPH, previous caesarean section and certain medical disorders (e.g. coagulopathy, diabetes mellitus, cardiovascular diseases). These risk indicators have an increased risk for PE, PPH or both, therefore possibly confounding the observed association. [8], [11], [19], [53] However the role of body mass index on PPH is not clear yet. [54] Another possible and not registered confounder is magnesium sulphate, since it could, hypothetically, increase blood loss by vasodilatation, a tocolytic effect predisposing to uterine atony, prolonged bleeding time through platelet activity inhibition and red cell deformity and it is used in the Netherlands for the management of (pre-) eclampsia (as prevention in severe PE women (defined as blood pressure ≥110 mmHg diastolic or ≥110 mmHg systolic or proteinuria ≥5 grams or the presence of symptoms prognostic for eclampsia) or as treatment in women with eclampsia). Rouse et al determined an increased risk for PPH in hypertensive disorders which disappeared after correction for magnesium sulphate. [55] However a recent published systematic review showed no significantly increased risk for PPH. [56]

### Findings in relation to other studies

**Association pre-eclampsia and postpartum haemorrhage.** Most other studies evaluate multiple risk indicators for PPH, in which PE is one of many. Results are however inconsistent, varying from no significant association after adjustment for confounders to a two to five fold increased risk [8], [11], [12], [14]–[16], [22], [37], [38], [55], [57]. Nevertheless, results are difficult to compare since various definitions for PPH are used in combination with different ways to estimate blood loss values. Only one study focused on the relation of PE and PPH. In a cohort study of 315 085 singleton pregnancies, Eskild et al showed an increased risk for PPH in women with PE (≥500 ml blood loss, OR 1.94 95% CI 1.87 to 2.02 and for ≥1500 ml blood loss OR 2.20, 95% CI 1.99 to 2.45) [21]. Similar results were found in nulliparous women with a vaginal mode of delivery. However they did not adjust for age, ethnicity, gestational age or socioeconomic status. Neither did they evaluate subgroups of women with and without induction of labour.

**Other risk indicators for postpartum haemorrhage.** Comparing our findings with other studies apart from PE as risk indicator, we see similar increased risks for older women, multiple pregnancy, birth weight ≥4000 grams, prolonged ruptured membranes and prolonged expulsive phase. [8], [11]–[16], [22], [36]–[39], [58]

In contrast with other studies, only great grand multiparas (para ≥10) had an increased risk for PPH (OR 1.57, 95% CI 1.12 to 2.20) when compared with multiparas (para 1–4). Other studies showed similar results, suggesting a possible linear relationship. [8], [59], [60]

An unexpected finding was the decreased risk for PPH after emergency caesarean sections. Other studies showed increased risks, which is more likely since this group often suffers from predisposing indicators for PPH, such as prolonged labour. [8], [15], [37], [57], [61] Possibly, emergency caesarean sections were misclassified as elective due to the intention to treat reporting of caesarean deliveries in the Netherlands. [30] In addition, PPH may be underreported in caesarean sections, [62] which can be expected more often in emergency situations. This could have confounded the risk of PPH in preeclampsia in our total group of women, however, the increased risk persisted in the subgroup-analysis of women who had non-induced spontaneous deliveries.

### Meaning of the study

This study indicates a strong relationship between the two most important causes of maternal mortality and morbidity worldwide: PE and PPH. This is an important finding in relation to the alarming notion that there is a rising incidence of both PE and PPH. Even though this study shows an efficient management for PE, the rising overall prevalence of PE and PPH indicates these problems have not been resolved. [17], [18], [20], [34] Optimising prevention and management of these conditions continues being of utmost importance. A higher awareness is indicated during the third stage of labour in a women with PE. Also other preventive and therapeutic measures should be considered, such as easy access to second-line uterotonics, materials for tamponade and packed cells.

Considering the complexity of PE with vascular changes and hemoconcentration in conjunction with the consequences of PPH leading to transfusion, embolisation or surgery, a combination of these two conditions forms a strong indication for a multidisciplinary approach, consisting of obstetric, anaesthetic, radiological and internal support.

### Future research

The most important question arising while studying the literature was the variation in risk of indicators for PPH. This can be attributed to the heterogeneity of all studies on PPH, consisting of varying study populations, definitions and statistical analyses performed. A uniform research definition is important to create comparable studies on PPH. With more homogenous studies prediction models for PPH could be formed, from which clinical implications can be drawn. Future research should therefore be on a universally acceptable definition and on prediction models for PPH with internal and external validations.

Further research in the pathogenesis of PE is also important in discerning the association of PE and PPH.

Finally, the observed trends in both PE and PPH should be investigated more detailed to discern whether the Dutch nationwide data can explain these discomforting findings. A study investigating trends for PPH in the Netherlands is currently in production.

## Conclusions

In conclusion, our study shows an association between PE and PPH; women with PE have a 1.53 fold increased risk for PPH in the Netherlands. Clinicians should be aware of this increased risk and use this knowledge in the management of PE and the third stage of labour in order to reach the fifth Millennium Developmental Goal.
